# Bilateral Lipoadenoma of the Adrenal Glands and Humeral Brown Tumor: An Unusual Association

**DOI:** 10.1155/2021/4870493

**Published:** 2021-08-27

**Authors:** N. Messaoudi, N. Bouichrat, M. Karrou, I. Assarrar, S. Malki, S. Elmahjoubi, S. Rouf, N. Karich, A. Lachkar, S. Sefiani, M. Bouziane, I. Kamaoui, H. Latrech

**Affiliations:** ^1^Department of Endocrinology-Diabetology and Nutrition, Mohammed VI University Hospital Center, Faculty of Medicine and Pharmacy, University of Mohammed 1st, Oujda, Morocco; ^2^Department of Anatomical Pathology, Mohammed VI University Hospital Center, Faculty of Medicine and Pharmacy, University of Mohammed 1st, Oujda, Morocco; ^3^Department of Orthopedic Surgery B, Mohammed VI University Hospital Center, Faculty of Medicine and Pharmacy, University of Mohammed 1st, Oujda, Morocco; ^4^AGDAL Anatomical Pathology Center, Rabat, Morocco; ^5^Department of General Surgery, Mohammed VI University Hospital Center, Faculty of Medicine and Pharmacy, University of Mohammed 1st, Oujda, Morocco; ^6^Department of Radiology, Mohammed VI University Hospital Center, Faculty of Medicine and Pharmacy, University of Mohammed 1st, Oujda, Morocco

## Abstract

Adrenal adenolipomas are rare lipomatous adrenal tumors that can be either functional or not. Only 7 cases have been reported in the English literature so far. However, brown tumors are benign, rare, historical lesions, with histological similarity to giant tumors that can be encountered in 1% of all primary hyperparathyroidism cases. We report the case of an unusual association of bilateral lipoadenoma of the adrenal glands and humeral brown tumor in a 35-year-old patient. He presented to the emergency department with a pathological fracture of the left humerus secondary to a brown tumor. The medical investigations have concluded to primary hyperparathyroidism. The screening for multiple endocrine neoplasia type 1 revealed the presence of bilateral nonsecreting adrenal masses whose anatomopathological study concluded adenolipomas. Adrenal tumors may constitute a part of multiple endocrine neoplasia type 1 in 20 to 40% of cases. In this view, it is necessary to check for the presence of other endocrine gland tumor locations such as primary hyperparathyroidism, neuroendocrine tumors of the duodenum and pancreas, or pituitary adenomas.

## 1. Introduction

Lipomatous tumors of the adrenal gland are a group of tumors that have in common a significant component of adipose tissue. They are usually benign and nonfunctional. Myelolipomas are the most frequently described entity in the literature; other tumors have also been described, such as teratomas, lipomas, angiomyolipomas, and exceptionally adrenal adenolipomas [1].

Adrenal adenolipomas are extremely rare lipomatous adrenal tumors that can be functional or not. They have been described for the first time in 1995 [2]. To our knowledge, only 7 cases have been reported in the English literature to date [1].

The association of adrenal adenolipoma and brown tumors has never been reported in the literature. This association may be part of a multiple endocrine neoplasia type 1 (MEN1). In this paper, we report the case of an unusual association of bilateral lipoadenoma of the adrenal glands and a humeral brown tumor in a 35-year-old patient.

## 2. Case Report

**Figure 1:**
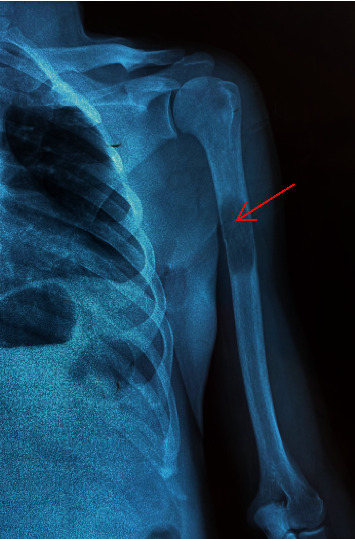
Standard X-ray showing a lytic lesion with cortical thinning and pathologic fracture in the left proximal humerus (red arrow).

A 35-year-old patient with a medical history of gout under dietary regimen and an undocumented dysthyroidism in the mother was admitted to the emergency room for the management of a fracture of the left humerus without prior trauma (Figure 1).

Considering the suspicious osteolytic image of the fracture site on the standard X-ray (Figure 1), a magnetic resonance imaging (MRI) scan of the left arm was performed to confirm the presence of a centromedullary osteolytic tumor process of the left humeral diaphysis, responsible for a pathologic fracture.

Malignant hypercalcemia at 4.72 mmol/l and then 4.49 mmol/l (normal range: 2.1 mmol/l–2.6 mmol/l using the colorimetric spectrophotometric method) was discovered on the preoperative biological assessment, requiring intravenous rehydration (500 ml of 0.9% saline solution every 4 hours, i.e., 3 l per day) and 3 hemodialysis sessions followed by an infusion of zoledronic acid 4 mg. The blood calcium level decreased at 2.61 mmol/l.

**Figure 2:**
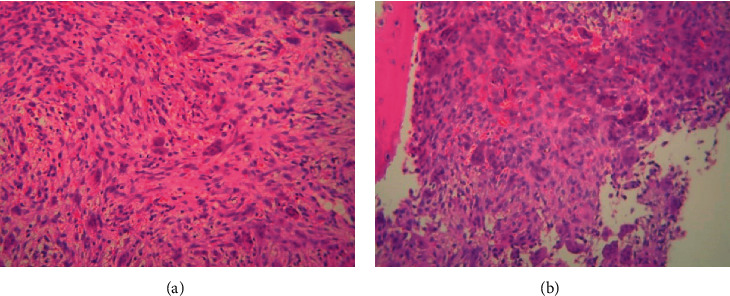
(a) Proliferation of fibroblasts mixed with giant cells (magnification: 100x). (b) Bone infiltration by a brown tumor (magnification: 100x).

The patient then underwent centromedullary nailing with bone biopsy. The anatomopathologic results showed a connective tissue and bone with thinned and rarefied trabeculae surrounded by osteoblasts (Figure 2(a)), associated with numerous multinucleated giant cells and siderophages (Figure 2(b)) compatible with a brown tumor.

The patient was then hospitalized in our endocrinology department for etiological assessment of his hypercalcemia.

The anamnesis found no functional signs, including the absence of previous bone pain or dental problems. The blood tests revealed a primary hyperparathyroidism made of hypercalcemia, a low phosphoremia at 17 mg/l (normal range: 24 mg/l–45 mg/l), a high biointact parathyroid hormone (iPTH) 1–84 at 795 pg/ml (normal range: 14.9 pg/ml–56.9 pg/ml), an elevated 24 h calciuria at 550 mg/24 h, and a low vitamin D at 7.8 ng/ml.

**Figure 3:**
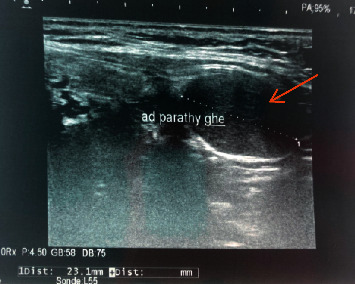
Cervical ultrasound showing an oval, well-defined, hypoechoic nodule, with regular contours, measuring 33 ∗ 32 ∗ 18 mm (red arrow).

**Figure 4:**
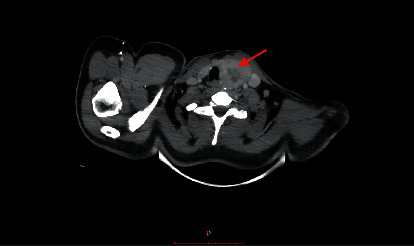
Cervicothoracic CT scan revealing a hypodense nodule in the posteroinferior pole of the left thyroid lobe (red arrow).

Cervical ultrasonography showed the presence of an oval nodule at the lower left parathyroid lodge that was bilobed, frankly hypoechoic with hypervascular Doppler signal, measuring 33 ∗ 32 ∗ 18 mm (Figure 3). The same parathyroid adenoma was located on the cervicothoracic computed tomography scan (Figure 4).

**Figure 5:**
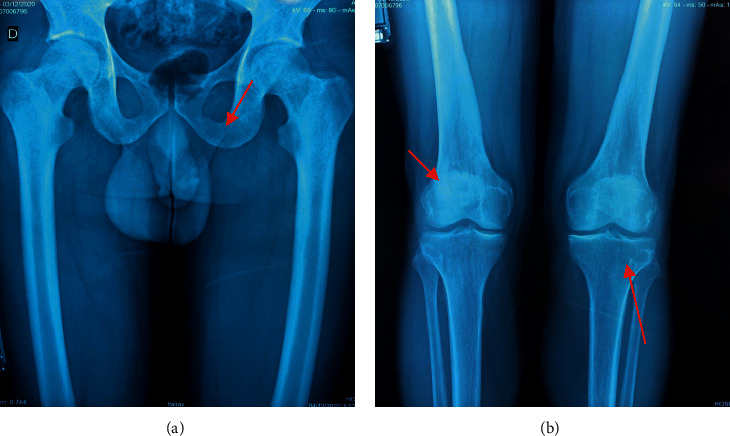
Radiographs showing multiple well-limited osseous lytic lesions with sclerotic margins (red arrows). (a) Ischiopubic ramus. (b) The distal extremity of the femur and the upper end of the tibia.

The evaluation of hypercalcemia impacts did not find any cardiac repercussions; however, the X-rays of the long bones showed multiple brown tumours in the ischiopubic ramus (Figure 5(a)), in the distal extremity of the femur and the upper end of the tibia (Figure 5(b)), and the pancreatic CT scan found small calcification of the head of the pancreas.

**Figure 6:**
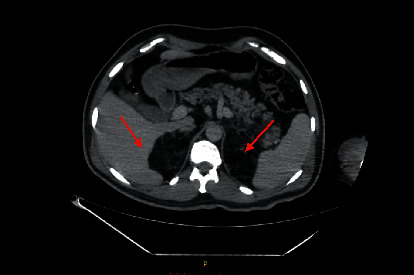
Abdominal CT scan showing bilateral multilocular adrenal masses, with a predominantly fat density (−75 UH), measuring 64 ∗ 59 ∗ 42 mm on the right gland and 102 ∗ 93 ∗ 69 mm on the left gland (red arrows).

In view of the young age of the patient, the search for forms that fit into the context of multiple endocrine neoplasia has been initiated. CT scan of the adrenal glands and pancreas revealed the presence of bilateral adrenal masses, with embossed, multilocular contours, and a predominantly fat density, measuring 64 ∗ 59 ∗ 42 mm on the right gland and 102 ∗ 93 ∗ 69 mm on the left one (Figure 6). Acute adrenal insufficiency was eliminated on the blood serum ionogram (potassium was at 3.3 meq/l, and sodium was at 134 meq/l). Biochemical screening for adrenal hypersecretion found normal plasma catecolamines, with neither hypercorticism nor hyperaldosteronism.

Brain imaging did not find any pituitary mass. The blood test revealed normal gonadotropin and prolactin levels.

Furthermore, a hyperthyroidism was noted in the thyroid assessment with negative TSH-receptor-antibodies and calcitonin. Cervical ultrasound showed an enlarged thyroid gland measuring 63 cc with multiple Thyroid Imaging Reporting and Data System (TI-RADS) 3 nodules in the right thyroid lobe and a TI-RADS 4 nodule with a long-axis diameter of 5 cm in the left thyroid lobe.

The rest of the evaluation did not find any bronchial or thymic tumors or additional pancreatic abnormalities.

The case was discussed over a multidisciplinary meeting. The decision was to initially perform a bilateral adrenal tumorectomy, followed by a lower-left parathyroidectomy and a total thyroidectomy. The pathological analysis of the adrenal specimen was in favor of adrenal adenomyolipomas without signs of malignancy. A 250 *μ*g Synacthen® test was performed. The cortisol level within 60 minutes reached 147 ng/ml.

**Figure 7:**
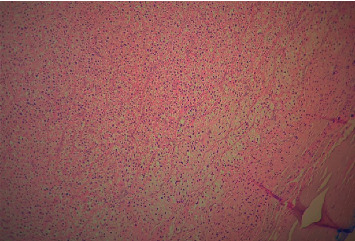
Magnification 100x, showing well-circumscribed tumor proliferation with thin fibrous capsule. Tumor cells are monotonous not atypical (HE: 100x).

**Figure 8:**
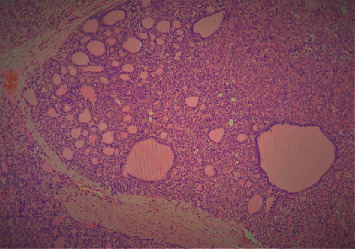
Magnification 100x, showing tumor proliferation of vesicular pattern with capsular invasion (HE: 100x).

Hypercalcemia was controlled by regular injections of zoledronic acid. Once normocalcemia was achieved, the patient underwent lower left parathyroidectomy with total thyroidectomy. The anatomopathological results confirmed the diagnosis of parathyroid adenoma without signs of malignancy (Figure 7). As for the thyroidectomy specimen, the histological study showed a vesicular carcinoma with capsular effraction and vascular emboli, classified pT3aNxMx (Figure 8), and a lymph node dissection with a radioiodine-131 therapy (RIT) will be scheduled.

The postoperative evolution noted a normalization of phosphocalcic markers. The calcemia reached 2.3 mmol/l, and iPTH reached 11 pg/ml (normal range: 9,2 pg/ml–44,6 pg/ml). The genetic testing is still ongoing.

## 3. Discussion

Primary adrenal tumors are rare, with a poorly elucidated prevalence, most often discovered incidentally. Some studies estimate that the frequency of adrenal tumors increases with age and may reach 10% of the elderly population [3]. Adrenal tumors are classified according to their location, the uni- or bilateral localisation, functional or unfunctional character, and their evolutive profile. The most frequently encountered etiologies are nonfunctional benign adrenocortical adenomas in first rank followed by adrenal myelolipomas.

Adrenal myelolipoma is the most frequent and most described lipomatous adrenal tumor in the literature. It represents 3% of all adrenal tumors. Much rarer, other lipomatous tumors of the adrenal cortex can be found, such as teratomas, angiomyolipomas, lipomas, and more rarely adenolipomas.

Adrenal adenolipomas are extremely rare lipomatous adrenal tumors, first described in 1995 [2]. They have had several names: adrenal cortical adenomas with fat component in 1995 [2], adenomas with lipomatous metaplasia [4], and lipoadenomas [5, 6]. To date, only 7 cases of unilateral adenolipomas have been described in the English literature including 5 cases of secreting adenolipomas (Table 1). In our patient, the adrenal tumors are bilateral, nonsecreting and of fortuitous discovery even with their large size.

Despite the small sample size reported in the literature, the average age of the patients was 47.8 years with a female predominance (sex ratio M/F: 2/5). The size of the mass varied from 20 mm to 100 mm with an average of 60 mm, and a higher frequency on the right side (57%).

Adrenal adenolipomas are the main differential diagnosis with adrenal myelolipomas. They have the same clinical and radiological characteristics: the only difference resides in the histological pattern, as adrenal adenolipomas do not comprise hematopoietic tissues or calcifications [6]. Adrenal tumors, especially those of the adrenal cortex, whether functional or not, could be a part of a MEN1 in 20 to 40% of cases, which is why it is mandatory to screen for other endocrine gland tumor locations. Our case presents the unique association of adrenal adenolipoma and primary hyperparathyroidism complicated by a brown tumor. All can be integrated into MEN1.

MEN1 is a rare genetic syndrome, first described in 1954 [9], also known as Wermen's syndrome [10], and is listed in the McKusik classification “OMIM 131100” [11]. It is defined by the association, in the same individual or in related persons belonging to the same family, of hyperparathyroidism, pancreatic or duodenal neuroendocrine tumors, pituitary adenoma, adrenal cortical tumors, and bronchial, thymic, or gastric neuroendocrine tumors. Other tumor proliferations of nonendocrine tissues can also be encountered, such as angiofibromas, lipomas, and collagenomas. Its prevalence is poorly known and is estimated at 25/100,000, affecting all age ranges from 5 to 82 years. The clinical (Table 2) and biological onset symptoms appear around the fifth decade in 94% of cases [10]. It can be sporadic in 10 to 14% of cases.

Hyperparathyroidism is the most common condition in MEN1 [9]. It is found predominantly in females and is fortuitously discovered in 75% to 80% of cases through a systematic measurement of blood calcium levels. The clinical signs are secondary to hypercalcemia: renal colic, bone pain, and pathologic fractures.

The bone manifestations are various, including fibrocystic osteitis, brown tumors, and pathological fracture. Brown tumors are benign, rare, historical lesions, with histological similarity to giant tumors. They result directly from the action of parathormone on the bone structure. The brown color is secondary to hemosiderin deposits. They are encountered in 1% of primary hyperparathyroidism cases [12]. Their radiological aspects include lytic lesions, with well-limited contours, without sclerosis, being either eccentric or cortical, with thinning, blowing, or rupture of the cortex [13]. In most cases, the brown tumors secondary to primary hyperparathyroidism are unique and are most often located in the facial bones, more rarely in the long bones, pelvis, spine, and base of the skull [14]. Only a few cases of multiple brown tumors have been reported in the literature [15]. In our case, the patient presented with multiple lytic lesions with cortical thinning and a pathologic fracture in the left proximal humerus.

The first step in the treatment of brown tumors is the management of primary hyperparathyroidism. Some authors report spontaneous resorption of these tumors after parathyroidectomy [16].

Our patient has 3 conditions that are part of MEN1: primary hyperparathyroidism, an adrenocortical tumor, and a vesicular carcinoma of the thyroid. The genetic study for the MEN1 mutation is currently ongoing. Due to the strong clinical presumption, the patient requires regular and strict surveillance given the risk of the appearance of other tumor conditions.

## 4. Conclusion

Adrenal adenolipomas are very rare tumors. Their diagnosis is based on histopathology. The current progress of imaging, the development of new strategies of hormonal evaluation, and histological analysis allow a better understanding and studying of their characteristics. In view of the frequency of associations that often take part of MEN1, the screening for other endocrine disorders is necessary when an adrenal mass is diagnosed, whatever its particularities. In order of frequency, a systematic dosage of the calcemia is needed in order to make an early diagnosis of hyperparathyroidism and to avoid the development of complications such as the brown tumors reported in our patient. To our knowledge, this particular association has never been described before in the existing literature.

## Figures and Tables

**Table 1:** Characteristics of adenolipomas described in the literature.

Reference	Age	Sex	Discovery circumstances	Localization	Size (mm)	Secretion
Sato et al. [2]	47	M	Abdominal pain	Left	35	Absent
Papotti et al. [4]	79	F	Abdominal pain	Left	60	Present
Papotti et al. [4]	46	F	Fortuitous	Right	110	Present
Uriev et al. [5]	51	F	Cushing's syndrome	Left	40	Present
Mylarappa et al. [7]	12	F	Hirsutism	Right	100^*∗*^80	Present
Martins et al. [8]	53	F	Cushing's syndrome	Right	28	Present
Luo et al. [6]	46	M	Abdominal pain	Right	20^*∗*^15^*∗*^13	Absent
Our case	35	M	Fortuitous	Bilateral	Right: 64^*∗*^59^*∗*^42 Left: 102^*∗*^93^*∗*^69	Absent

**Table 2:** Clinical manifestations of MEN1 syndrome.

Clinical manifestations	Prevalence (%)
Hyperparathyroidism	90
Neuroendocrine tumors of the duodenum and pancreas	Nonfunctioning: 55
	Gastrinoma: 40
	Insulinoma 10
	Glucagonoma <1
	VIPoma <1
Pituitary adenomas	40
Adrenocortical tumors	20–40
Pheochromocytomas	<1
Neuroendocrine thymic tumors	2
Bronchopulmonary neuroendocrine tumors	5
Angiofibromas	85
Collagenomas	70
Lipomas	30
Other tumors	
Meningioma	8
Ependymoma, melanoma, thyroid	0–25

## Data Availability

The patients' data used to support the findings of this study can be retrieved from the archives of the Department of Endocrinology-Diabetology and Nutrition, Mohammed VI University Hospital, Oujda, Morocco.
